# Smoking cessation during COVID-19: the top to-do list

**DOI:** 10.1038/s41533-021-00238-8

**Published:** 2021-05-06

**Authors:** Jaber S. Alqahtani, Abdulelah M. Aldhahir, Tope Oyelade, Saeed M. Alghamdi, Ahmad S. Almamary

**Affiliations:** 1 UCL Respiratory, University College London, London, UK; 2 Department of Respiratory Care, Prince Sultan Military College of Health Sciences, Dammam, Saudi Arabia; 3 Respiratory Care Department, Faculty of Applied Medical Sciences, Jazan University, Jazan, Saudi Arabia; 4 UCL Institute for Liver and Digestive Health, London, UK; 5 National Heart and Lung Institute, Imperial College London, London, UK; 6 Faculty of Applied Medical Sciences, Umm Al-Qura University, Makkah, Saudi Arabia; 7 Respiratory Therapy Department, King Saud Bin Abdulaziz University for Health Sciences, Ahsa, Saudi Arabia

**Keywords:** Patient education, Health care

## Abstract

As evidence continues to emerge, our understanding of the relationship between smoking and COVID-19 prognosis is steadily growing. An early outlook from World Health Organisation (WHO) indicates that smokers are more vulnerable to severe COVID-19 disease and are also more likely to be infected, as frequent motions from hand to mouth and sharing of tobacco products such as waterpipes increased the possibility of being infected. In this commentary, we discuss some of the latest evidence on smoking and COVID-19 and emphasise the need to promote the personal and public advantages of smoking cessation during the COVID-19 pandemic.

## Smoking and COVID-19 risk

Every year, smoking kills >8 million people worldwide. Of such deaths, over 7 million are directly related to cigarettes, and ~1.2 million are attributed to second-hand smoke by non-smokers^1^. No doubt, that smoking is a major risk factor for most respiratory-related infections, which raises the severity of respiratory diseases. The early evidence in this area found that compared with non-smokers, smokers are more likely to develop serious COVID-19 disease^2,3^. In a study carried out by Mehra et al.^4^ of over 8910 COVID-19 patients across three continents namely Asia, Europe, and North America, current smoking when compared to former or never smoke was an independent risk factor for death at hospital with odds ratio of 1.79 (95% confidence interval: 1.29–2.47). Indeed, smoking is heavily contributing to the progression of such long-term diseases, including cardiovascular and respiratory^5^.

Recently, accumulated evidence from the largest meta‐analysis among peer‐reviewed literature that included 32,849 hospitalised patients with COVID‐19 found that current smokers had an increased risk of severe COVID‐19 (risk ratios: 1.80; 95% confidence interval: 1.14–2.85)^6^. Even though smoking might not generally raise the risk of contracting COVID-19, it may be particularly debilitating for a smoker if there is a biological and inflammatory cascade, following a SARS-CoV-2 infection. Recent evidence suggests a low prevalence of active smokers among those COVID-19 patients who need hospital admission in many countries severely affected by COVID-19 pandemic^7,8^. Such underrepresentation of smokers among those patients supported claims that smoking could be protective from COVID-19 infection. Possible mechanisms can support this claim: nicotine anti-inflammatory effect, a blunted immune response in current smokers and potential high nitric oxide in the upper respiratory airway, which may prevent replication of SARS-CoV-2 and its entry into cells^9^.

As scientists get more evidence in this area, more concerns arise about other smoking products, such as vaping. Considering the risks of significant pulmonary injury with vaping^10^, McAlinden et al. hypothesise that similar risks may happen to COVID-19 patients who are vaping^11^. The potential risk of vaping to prime the lung for SARS-CoV-2 is speculative, given that vaping prevalence in COVID-19 patients has not been reported. However, what we know already is that older age groups are the ones more likely to develop severe COVID-19, and according to survey studies on e-cigarette use in China and the USA, vaping is not prevalent in those of 65 years and older, but popular in younger groups 15–44 year age range^12,13^. Indeed, like smoking, it is likely, if it is not a risk factor for contracting COVID-19, that vaping may also lead to bad outcomes.

## Why smoking causes more severe COVID-19 cases

Several hypotheses exist regarding smokers tend to have worse outcomes after contracting COVID-19. Angiotensin-converting enzyme 2 (ACE-2)—the only confirmed SARS-CoV-2 receptor, is a vasodilatory protein expressed on the surface of the lung cells and serves as the cell-entry receptor for SARS viruses^14^. Recent evidence showed that compared with former and never smokers, smokers were associated with higher ACE-2 expression in a various cohort of lung tissue and airway epithelium cells^15–17^. Such an observation was further supported by other studies linking nicotine exposure, with increased ACE-2 expression^18,19^. Thus, it could be speculated that the increased ACE-2 expression in current smokers may perhaps predispose to increased risk of SARS-CoV-2 infection. However, it is important to note that to date there is no direct evidence linking increased expression of ACE-2 to increased susceptibility or severity to COVID-19.

**Fig. 1:**
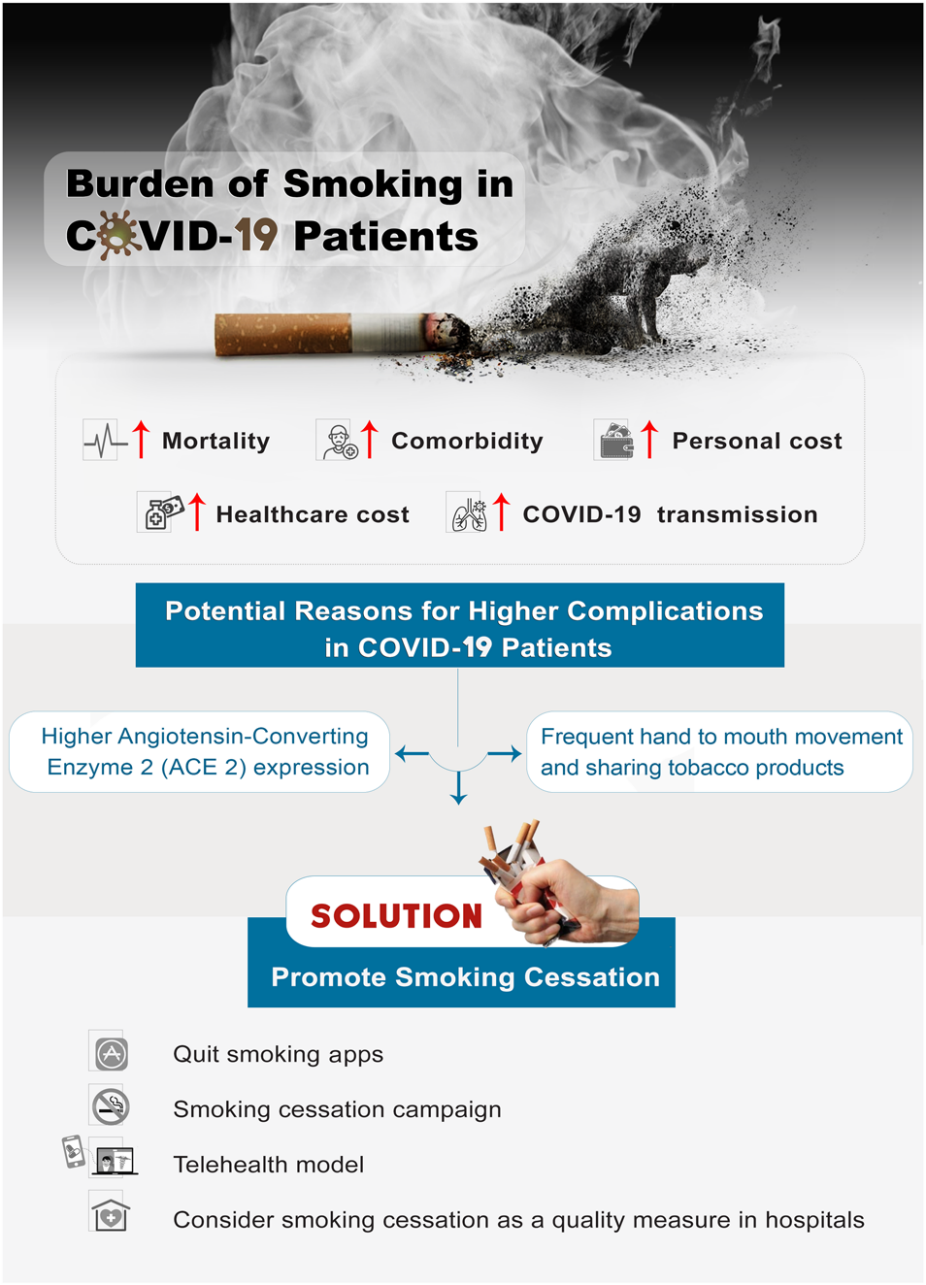
Burden of smoking in COVID-19 patients and role of smoking cessation. Smoking is associated with increased risk of severe COVID-19 and mortality that are linked with ACE 2 overexpression and frequent hand to mouth movement. The health cost of COVD-19-associated morbidity can be reduced via the promotion of smoking cessation through apps, public health campaigns, telehealth models and the hospital quality measures.

Despite the high prevalence of smokers in Italy (25.7%), Rossato et al.^20^ reported a very low prevalence of smokers in COVID-19 patients, with no significant association between smokers and severe disease in COVID-19 patients. According to Leung et al.^21^, the potential explanation for this low prevalence could be smoking status misclassification due to underreporting of smoking in such cohorts or perhaps because of using some medications that may offer some protection against COVID-19. Thus far, there is no evidence that smoking protects against COVID-19. Indeed, the speed of events during COVID-19 makes it quite challenging to verify the truth from false evidence. Therefore, it is crucial to be extremely careful about the messages about smoking and COVID-19 in these fragile times. Figure 1 shows the burden of smoking in COVID-19 patients.

## How can we support smoking cessation during the COVID-19 pandemic?

### Why smokers should quit?

With over 8,000,000 deaths per year due to direct or indirect exposure, smoking remains a major global health issue especially in low- and middle-income countries. Indeed, in the context of COVID-19, this number is expected to go up, but the time has not gone by to quit smoking. With an average light smoker consuming 10–15 cigarette/day, including an average of up to 13 puffs per cigarette and each puffs duration of 1.5 s (refs. ^22,23^). This shows that an average smoker has a hand to their mouth between 130 and 195 times. This translates into 195–293 s more, in which the hand is on the lip per day compared with non-smokers. This is especially interesting giving that the SARS-CoV-2 can survive on surfaces for days depending on the medium of shedding. This provides a possible hypothesis for the increased risk of SARS-CoV-2 infection and/or re-infection in smokers compared to non-smokers. Therefore, both smokers and healthcare providers should be aware of this practice behaviour. According to a recent study, many smokers showed substantial levels of interest expressed in accessing various forms of cessation assistance, during COVID-19 compared before COVID-19 (ref. ^24^). This shows that smokers understand how to reduce the risk of COVID-19 by addressing their smoking. Action on Smoking and Health (Ash) calculates that around a million smokers stopped smoking during COVID-19, an additional 550,000 have attempted to quit and 2.4 million have been reduced to smoke cigarettes, which COVID-19 provided us an unexpected opportunity to help more people quit smoking^25^. Put together, the COVID-19 pandemic and the relative risk of severity linked with smoking furthermore justifies the need for a unified and global public health campaign targeted at discouraging direct or indirect tobacco exposure around the world.

### Health and financial benefits

We highly advocate providing country-specific, evidence-driven smoking cessation recommendations in public health communications focusing on how to curb the spread of SARS-CoV-2. Such cessation attempts can provide a variety of advantages within and after the present pandemic, including a reduction in the incidence of smoking-related conditions, such as myocardial infarction and chronic respiratory conditions, and ultimately reduce the clinical challenge faced by frontline clinicians^26–28^. On top of that, there are personal and healthcare system financial savings because of smoking cessation. Smoking has many detrimental consequences on cardiovascular and respiratory functions, which high-quality research shows that preoperative reduction of smoking by using smoking cessation interventions can lead to substantial health benefits^29^. Smoking cessation is a significant public health gain at any moment. A prospective study included 1.3 million participants, which resurveyed postally ~3 and 8 years later found that two-thirds of all deaths of smokers in their 50s, 60s, and 70s are caused by smoking; smokers lose at least 10 years of lifespan^30^. They also demonstrated that ceasing before age 40 years prevents >90% of the additional mortality triggered by continuing smoking; quitting before age 30 years avoids >97% of it.

### Policy and practice

At this time during COVID-19 pandemic, it is the right time that the ongoing concern about the COVID-19 outbreak undoubtedly offers a ‘learnable window’, when smokers will be exclusively open to smoking consultation for smoking cessation. This has been seen in a recent study that found the rate of smoking cessation increased from 23% before COVID-19 to 31% during COVID-19 pandemic^31^. Such health and financial benefits are maximised by psychological and pharmacological interventions that delivered from specialist smoking cessation services^32^. However, smoking cessation services should be more resilient and change their traditional delivery models in response to COVID-19 by using telehealth tools, such as expanding telecommunications and interactive assistance capabilities, and allow remote access to nicotine replacement products^33^. Further, promoting tech-driven approach by using quitting smoking apps is more beneficial to increase the odds of quitting success. A randomised clinical trial published recently compared the efficacy of two smartphone applications for smoking cessation, and found that smokers who used the iCanQuit app (teaches acceptance of smoking triggers) had 1.49 times higher odds of quitting smoking compared with those who used the QuitGuide app (depends on avoiding smoking triggers)^34^. This elevates apps as an effective way of quitting smoking, particularly apps that depend on accepting the existence of cravings rather than trying to eliminate them.

Indeed, using such strategies would reduce the smoking burden on healthcare systems. A recent study synthesised that best hospitals should be required to deliver smoking cessation, and such a service provision should be considered as a quality measure for accreditation and recognition^35^. Furthermore, policymakers and healthcare providers should consider the information delivery preferences of smokers when campaigning about smoking cessation (see Fig. 1). A recent study reported that most of the smokers preferred to receive such information through a television channel (61%), followed by online news (36%), social media (31%), and email (31%)^24^. Indeed, policymakers should establish innovative strategies to increase the uptake to the smoking cessation therapies, and eventually raise the chances of quitting to promote greater patients and system outcomes.

## Conclusion

Beyond the health and financial benefits of avoiding tobacco use before COVID-19 pandemic, an increase in quit rates may help to minimise public SARS-CoV-2 transmission and the associated severity with current smokers. Enhancing smoking cessation trials during this pandemic is a clinical priority and should be thoroughly endorsed. Smoking cessation with high-quality advice using unconventional approaches during respiratory virus epidemics, like COVID-19, should be part of public health efforts.

## Data Availability

No data were generated in the preparation of this manuscript.
